# To Wait or Not to Wait—Separate Mechanisms in the Oculomotor Circuit of Basal Ganglia

**DOI:** 10.3389/fnana.2017.00035

**Published:** 2017-04-11

**Authors:** Masaharu Yasuda, Okihide Hikosaka

**Affiliations:** Laboratory of Sensorimotor Research, National Eye Institute, National Institutes of HealthBethesda, MD, USA

**Keywords:** saccade, reward, substantia nigra pars reticulata, superior colliculus, monkey

## Abstract

We reach a goal immediately after detecting the target, or later by withholding the immediate action. Each time, we choose one of these actions by suppressing the other. How does the brain control these antagonistic actions? We hypothesized that the output of basal ganglia (BG), substantia nigra pars reticulata (SNr), suppresses antagonistic oculomotor signals by sending strong inhibitory output to superior colliculus (SC). To test this hypothesis, we trained monkeys to perform two kinds of saccade task: Immediate (visually guided) and delayed (visually-withheld but memory-guided) saccade tasks. In both tasks, we applied one-direction-reward (1DR) procedure to modify the level of goal-reaching motivation. We identified SNr neurons that projected to SC by their antidromic activation from SC. We stimulated SC on both sides because SNr neurons projecting to the ipsilateral SC (ipsiSC) and those projecting to the contralateral SC (contraSC) might have antagonistic functions. First, we found that ipsiSC-projecting neurons were about 10 times more than contraSC-projecting neurons. More importantly, ipsiSC-projecting SNr neurons were roughly divided into two groups which would control immediate and delayed saccades separately. The immediate-type SNr neurons were clearly inhibited by a visual target on the contralateral side in both visual- and memory-1DR tasks. The inhibition would disinhibit SC neurons and facilitate a saccade to the contralateral target. This is goal-directed in visual-1DR task, but is erroneous in memory-1DR task. In contrast, the delayed-type SNr neurons tended to be excited by a visual target (especially on the contralateral side), which would suppress the immediate saccade to the target. Instead, they were inhibited before a delayed (memory-guided) saccade directed to the contralateral side, which would facilitate the saccade. ContraSC-projecting SNr neurons were more variable with no grouped features, although some of them may contribute to the saccade to the ipsilateral target. Finally, we found that some ipsiSC-projecting SNr neurons were inhibited more strongly when reward was expected, which was associated with shortened saccade reaction times. However, many SNr neurons showed no reward-expectation effect. These results suggest that two separate oculomotor circuits exist in BG, both of which contribute to goal-directed behavior, but in different temporal contexts.

## Introduction

Eye movement guides one’s body toward a goal. In ecological environment the resources are limited; therefore, animals (or humans) should reach a goal promptly once they find it. However, the goal is sometimes unreachable and they need to withhold the action for a while. These two actions are conflicting because immediate action does not give the animal enough time to examine if the target is reachable or not. To control these antagonistic actions, the brain should hold two separate mechanisms competing with each other.

The basal ganglia (BG) can contribute to the selection of behavior because their outputs are powerfully inhibitory and can suppress antagonistic actions (Mink, 1996). A prominent mechanism of the BG is to select a saccadic eye movement (Hikosaka et al., 2000, 2014) which guides us to reach a valuable object (Land and Hayhoe, 2001). This is performed by the inhibitory connection from the substantia nigra pars reticulata (SNr) to the superior colliculus (SC) in macaque monkeys (Hikosaka and Wurtz, 1983b). SNr neurons can thus facilitate a saccade by reducing the inhibition and suppress other saccades by enhancing the inhibition. Indeed, this selection mechanism may be at work, since SNr neurons increase and/or decrease their activity during a reward-biased saccade task (Sato and Hikosaka, 2002). However, it was not shown whether those signals were transmitted from SNr to SC.

Another elusive issue is the crossed connection of the SNr-SC pathway. Anatomical studies have shown that a small proportion of SNr neurons project to the contralateral side of SC, while many SNr neurons project to the ipsilateral side of SC (Jayaraman et al., 1977; Beckstead et al., 1981; Gerfen et al., 1982; Rhoades et al., 1982; Huerta et al., 1991). In terms of uncrossed connection, Hikosaka and Wurtz (1983a) examined the neuronal signal of SC-projecting SNr neurons, focusing on their inhibitory responses during visual and memory-guided saccade tasks. Our recent study showed that the uncrossed SNr-SC pathway operates to choose or avoid visual objects by their reward-associated values (Yasuda et al., 2012). However, the crossed SNr-SC pathway may work differently, according to Jiang et al. (2003).

In the present study, we first identified neurons in the monkey SNr that had either uncrossed or crossed connection to SC using antidromic stimulation from both sides of SC. We then addressed the main question described above by using two saccade tasks: (1) immediate (visually-guided) saccade task; and (2) delayed (memory-guided) saccade task. We found that the SNr neurons that had uncrossed connections to SC (uncrossed type) were largely separated into two groups: (1) neurons that facilitated immediate saccades by reducing their activity immediately in response to contralateral visual stimuli; and (2) neurons that suppressed immediate saccades by increasing their activity first, but facilitated delayed saccades by reducing their activity later when the goal became reachable. On the other hand, the SNr neurons with crossed connections (crossed type) showed various response patterns and may work in different and unknown conditions. These results suggest that antagonistic neuronal signals in the uncrossed SNr-SC pathway enable multiple actions to reach the goal immediately or lately.

## Materials and Methods

### Animals

Three rhesus monkeys (*Macaca mulatta*), N (male, 8 year/old, 11 kg), D (male, 8 year/old, 9 kg) and G (male, 10 year/old, 11 kg), were used as subjects in this study. All animal care and experimental procedures were approved by the National Eye Institute and Institutional Animal Care and Use Committee and complied with the Public Health Service Policy on the humane care and use of laboratory animals.

### Behavioral Task

Behavioral tasks were controlled by a QNX-based real-time experimentation data acquisition system (REX, Laboratory of Sensorimotor Research (LSR), National Eye Institute (NEI), National Institutes of Health (NIH), Bethesda, MD, USA). The monkey sat in a primate chair, facing a frontoparallel screen 33 cm from the monkey’s eyes in a sound attenuated and electrically shielded room. Stimuli generated by an active matrix liquid crystal display projector (PJ550, ViewSonic) were rear-projected on the screen. The monkey’s eye position was monitored by using the magnetic search coil technique. Saccadic eye movement was detected when the velocity of eye movement exceeded a threshold level (50°/s).

The monkeys were trained to perform two directional visually or memory-guided saccade task under one direction-large-rewarded condition (1DR; Kawagoe et al., 1998; Figure 2). In the present study, we used relative reward bias (large vs. small) instead of absolute reward bias (reward vs. no reward). In both tasks, monkey was forced to make a saccade to a cue position, which was randomly chosen from two possible opposite locations. Each trial started with the appearance of a central white spot which required the monkey to fixate on. After the fixation for 1000–1500 ms (fixation period), in visual 1DR, the central spot disappeared and a peripheral white spot was presented. The monkey was required to make a saccade toward the peripheral spot within 700 ms. In memory 1DR, after the fixation period, the peripheral spot of visual cue was briefly presented for 50 ms. The monkey had to maintain fixation after cue onset. When the central spot disappeared 1 s after the cue onset, the monkey had to make a saccade toward the remembered location of peripheral cue within 700 ms. In both tasks, during consecutive 24 trials, the monkey obtained large reward after saccade to one of two directions, but obtained small reward to the other direction. In next consecutive 24 trials, reward-direction contingency was reversed. During the recording these two blocks were alternatively repeated at least twice.

### Electrophysiology

Based on a stereotaxic atlas (Saleem and Logothetis, 2007), we placed two rectangular chambers in each monkey. We aimed at SNr from the lateral chamber and SC from the posterior chamber. The lateral chamber was placed over the fronto-parietal cortex, tilted laterally by 35°. The posterior chamber was placed over the midline of the parietal cortex, tilted posteriorly by 40°. MR images (4.7T, Bruker) were then obtained along the direction of the recording chamber which was visualized with gadolinium that filled grid holes and inside the chamber. Single-unit recordings and electrical stimulations were performed using tungsten electrodes (Frederick Haer) that were advanced by an oil-driven micro-manipulator (MO-97A, Narishige). The recording and stimulation sites were determined by using a grid system, which allowed recordings at every 1 mm between penetrations. In each daily experiment we introduced these electrodes into the brain, each through a stainless steel guide tube, which was inserted into one of the grid holes and then to the brain via the dura. For finer mapping of neurons, we also used a complementary grid, which allowed electrode penetrations between the holes of the original grid. The electrical signal from the electrode was amplified with a band-pass filter (200 Hz–5 kHz; BAK, Mount Airy, MD, USA) and collected at 1 kHz. Spike potentials of single neurons were isolated on-line using a custom voltage-time window discrimination software (MEX, LSR/NEI/NIH).

### Identification of SNr-SC Connections

To test if an SNr neuron projects its axon to SC, we used the antidromic activation method by electrically stimulating SC (Hikosaka and Wurtz, 1983b; Yasuda et al., 2012). In most of the recording sessions, we placed two stimulating electrodes in the left and right sides of SC. To position the SC electrode, we lowered the SC electrode until pre-saccadic activity was recorded. After switching the SC electrode from recording to stimulation, we lowered another electrode into SNr. To find a SC-projecting SNr neuron, we alternately stimulated both sides of SC until spikes with a fixed latency were detected. The antidromic nature of the spikes was confirmed using a collision test (Hikosaka and Wurtz, 1983b). For stimulation, we used a biphasic pulse with cathodal and anodal components, but the duration of each pulse was 100 μs.

### Data Analysis

To quantify neuronal response, we first calculated SNr neuron’s “pre-cue baseline activity”, “post-cue activity”, “pre-saccadic baseline activity”, and “saccadic activity” by counting the numbers of spikes within test windows. We set the baseline test windows for 200 ms before cue onset for “pre-cue baseline activity”, 200 ms from 100 ms to 300 ms after cue onset for “post-cue activity”, 300 ms from 600 ms to 300 ms before saccade onset for “pre-saccadic baseline activity”, and 300 ms from 200 ms before to 100 ms after saccade onset for “saccadic activity”.

The tasks contain four groups of trials: cue was presented on contralateral side to recorded hemisphere and large-rewarded trials (CL), cue-on ipsilateral and small-rewarded (IS), cue-on contralateral and small-rewarded (CS), and cue-on ipsilateral and large-rewarded (IL). By comparing neuronal responses between these four trial types, we analyzed neuronal discrimination for direction and reward. The directional and reward discriminations were defined as the area under the receiver operating characteristic (ROC) based on SNr neuron’s activity (“post-cue activity” or “saccadic activity”) in CL&CS trials vs. IL&IS trials and CL&IL trials vs. CS&IS trials, respectively. We also assessed neuronal discrimination for decrease/increase response in post-cue and saccadic period, which were defined as ROC based on “post-cue activity” vs. “pre-cue baseline activity” (in CL&CS trials), and “saccadic activity” vs. “pre-saccadic baseline activity” (in CL&CS trials) test window, respectively.

## Results

### Asymmetry between Uncrossed and Crossed Connection

**Figure 1:**
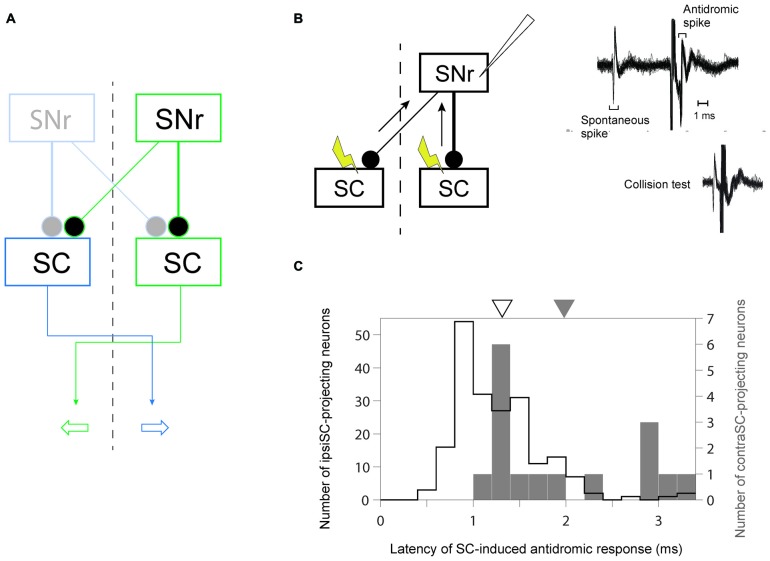
**Identification of uncrossed and crossed connections in substantia nigra pars reticulata (SNr)-superior colliculus (SC) pathway. (A)** A schematic diagram of the connections from SNr to SC. Since the projection from SNr to SC is inhibitory, the inhibition (excitation) of ipsiSC- and contraSC-projecting SNr neurons enhances (suppresses) contralateral and ipsilateral saccade, respectively. **(B)** Antidromic activation of an SNr neuron to electrical stimulation of the ipsilateral or contralateral SC. The antidromic nature of the spikes was confirmed by a collision test (right, bottom). **(C)** Faster signal processing in ipsSC- than in contraSC-projecting SNr neurons. The distribution of the latencies of SC-induced antidromic responses in SNr neurons. The mean of latencies in ipsiSC-projecting SNr neuron (white arrow: 1.3 ms) was significantly faster than that in contraSC-projecting SNr neuron (black arrow: 2.0 ms; *P* = 0.001, *t* test).

Our goal was to understand how the BG are involved in goal-directed saccade. To this end, it is crucial to identify the output oculomotor signal of BG. By stimulating both sides of SC, we recorded antidromically activated spike activity in SNr neurons which send their axons to SC in the same (ipsiSC-projecting SNr neuron) or/and the other side of hemisphere (contraSC-projecting SNr neuron; Figure 1B).

Previous anatomical studies reported that the number of contraSC-projecting SNr neurons were small and distributed more sparsely than ipsiSC-projecting SNr neurons (Jayaraman et al., 1977; Beckstead et al., 1981; Huerta et al., 1991). Our results confirmed this. Table 1 shows the number of SNr neurons that projected to SC for three monkeys. In all three monkeys, a majority of antidromically activated neurons projected to the same side of SC. We also measured the latency of antidromic response induced by SC stimulation. As shown in Figure 1C the latencies of ipsiSC-projecting SNr neurons were significantly shorter than contraSC-projecting neurons (*P* = 10^−3^, *t* test, uncrossed: mean ± SE = 1.3 ± 0.05 ms, crossed: mean ± SE = 2.0 ± 0.2 ms). This result suggests that ipsiSC-projecting SNr neurons can influence oculomotor command more quickly than contraSC-projecting SNr neurons.

**Table 1:** **The number of substantia nigra pars reticulata (SNr) neurons whose projection to superior colliculus (SC) were identified by antidromic stimulation**.

	ipsiSC	contraSC
Monkey N	74	5
Monkey D	61	8
Monkey G	63	3
Total	198	16

*For three monkeys, the number of contraSC-projecting SNr neurons are consistently lower than ipsiSC-projecting SNr neurons*.

### Separate Signal Processing for Goal-Directed Saccade in ipsiSC-Projecting SNr Neurons

The neurons in the intermediate layer of SC project to the other side of reticular formation to modulate the saccadic eye movement toward the contralateral direction (May, 2006). Consistent with the anatomical data, electrical stimulation (Robinson, 1972) and recording studies (Wurtz and Goldberg, 1972) showed that the activation of SC neurons enhances the saccade to the contralateral side. Therefore, an SNr neuron that projects to the same side of SC (ipsiSC-projecting SNr neuron) is likely to influence the saccade toward the position contralateral to the SNr neuron (contralateral saccade). In contrast, an SNr neuron that projects to the other side of SC (contraSC-projecting SNr neuron) is likely to influence the saccade toward the position ipsilateral to the SNr neuron (ipsilateral saccade; Figure 1A).

**Figure 2:**
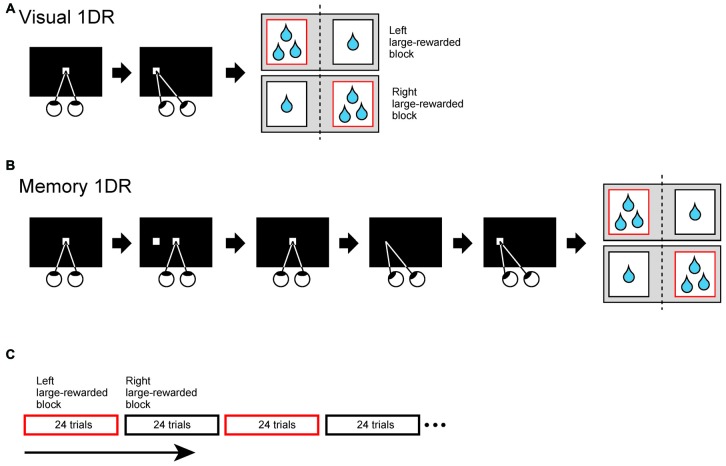
**Two directional visually (A)** or memory-guided **(B)** saccade task under one direction-large-rewarded condition (1DR). **(A)** Visually-guided 1DR. After fixating on a central white spot for 1000–1500 ms, the central spot disappeared and a peripheral white spot (cue) was presented. The monkey was required to make a saccade to the cue to obtain reward within 700 ms. **(B)** Memory-guided 1DR. After the fixation for 1000–1500 ms, the cue was briefly presented for 50 ms. The monkey had to maintain fixation after cue onset. When the central spot disappeared 1 s after the cue onset, the monkey had to make a saccade toward the cued location within 700 ms. **(C)** Trial schedule of the tasks. In both tasks, the amount of reward is dependent on the trial type. The monkey obtained large reward after saccade to one direction, but obtained small reward to the opposite direction. Large (small)-rewarded direction was fixed in consecutive 24 trials, but reward-direction contingency was reversed in next 24 trials. During the recording these two blocks were alternatively repeated at least twice.

To examine how SC-projecting SNr neurons encode directional and motivational signal to achieve goal-directed behavior, we trained three monkeys to perform saccade tasks under one direction-large-rewarded condition (1DR; Kawagoe et al., 1998). To separately examine immediate and delayed action signals, we prepared two task conditions: the one in which saccade was immediately driven by a visual cue (visual-1DR, Figure 2A), and the other one in which the saccade was internally driven based on the remembered position of the visual cue (memory-1DR, Figure 2B). The monkey’s motivation was modulated by changing the reward amount. In one block of 24 trials, the monkey received a large (or small) reward after making a saccade to one side (left or right). In the next block, the saccade-reward contingency was reversed (Figure 2C). This sequence was repeated at least twice.

**Figure 3:**
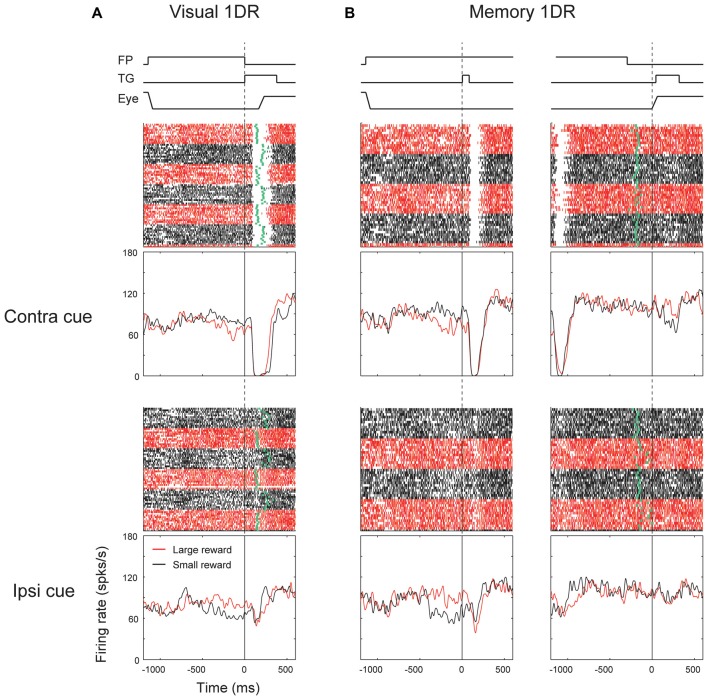
**Responses of an ipsiSC-projecting SNr neuron (immediate type) in visual (A)** and memory 1DR **(B)**. Raster plots and spike density functions (SDF) are shown in red (large-rewarded trials) and black (small-rewarded trials). Green tick in each raster line in **(A)** indicates the onset of the saccade toward the target (TG). Green tick in **(B)** indicates the offset of the fixation point (FP). Raster plots and SDFs were aligned on cue (**A** and left panels in **B**) or saccade (right panels in **B**) onset. The lines on the top shows the temporal sequence of events for each task. The activity in contra and ipsilateral saccade trials were shown in upper and lower panels, respectively.

We found that many ipsiSC-projecting SNr neurons showed a phasic inhibition after the visual cue presentation. Figure 3 shows the response of an ipsiSC-projecting SNr neurons. The neuron was strongly inhibited by the visual cue in both task conditions. The strong inhibition occurred only when the cue was presented on the contralateral side. The inhibition of the SNr neuron would cause a disinhibition of SC neurons on the ipsilateral side (Hikosaka et al., 2000), and therefore would facilitate saccades to contralateral side. This actually occurred in visual-1DR task (Figure 3A): the saccade started after the inhibition of the SNr neuron (green raster dots). While the saccade reaction time was clearly modulated by the reward amount, the activity of the SNr neuron showed no difference (overlapping black with red curves). Instead, the neuronal signal was separated before cue onset, suggesting that the SNr neuron may have contributed to the directional bias of saccade before the target appeared (Lauwereyns et al., 2002).

The strong inhibition signal after the target onset was not reflected in saccade initiation in memory-1DR (Figure 3B). In memory-1DR, the inhibitory response occurred after the visual cue onset when the monkey had to suppress saccades (Figure 3B, left). Moreover, the neuron’s activity showed no change before the memory-guided saccade (Figure 3B, right). These results suggest that this group of SNr neurons can play functional role in saccade initiation, but in a selective context: only when the saccade is caused immediately (i.e., visual-1DR), but not when the saccade is delayed (memory-1DR). Hereafter, we call this group of SNr neurons “immediate type”. Notably, monkeys sometimes made “fixation break error” after the cue onset in memory-1DR. This unwanted reflexive saccade may be caused by the inhibitory visual response of the immediate-type SNr neurons.

**Figure 4:**
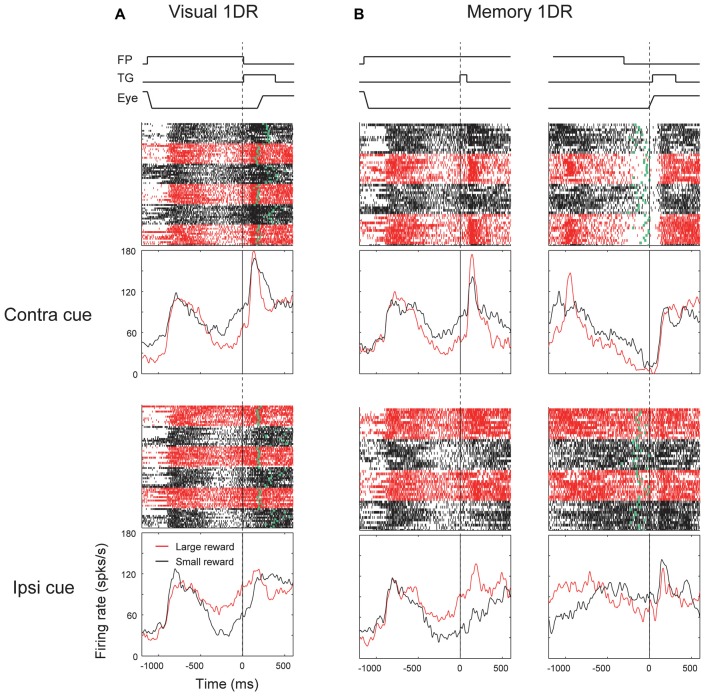
**Responses of an ipsiSC-projecting SNr neuron (delayed type) in visual 1DR (A)** and memory 1DR **(B)**. The same format as in Figure 3.

We found another group of ipsiSC-projecting SNr neurons. Figure 4 shows an example neuron. It was clearly excited after the cue onset in both visual-1DR (Figure 4A) and memory-1DR (Figure 4B), again selectively to the contralateral side. In contrast, the neuron was clearly inhibited before the saccade to the contralateral side during memory-1DR (Figure 4B, top-right). The inhibition was stronger in large reward trials (red vs. black: ROC = 0.11, *P* = 2.1 × 10^−6^, rank-sum test), which may contribute to the bias in saccade reaction time. The excitation after cue onset can contribute to the suppression of the immediate contralateral saccade, which is necessary in memory-1DR, but is invalid in visual-1DR. Moreover, the excitation started before cue onset especially when the contralateral saccade was to be followed by a small reward, which may also contribute to the suppression of the immediate contralateral saccade. These results suggest that this group of SNr neurons facilitate saccades specifically in the delayed context in memory-1DR. Therefore, we call the neurons with this response pattern “delayed type”.

**Figure 5:**
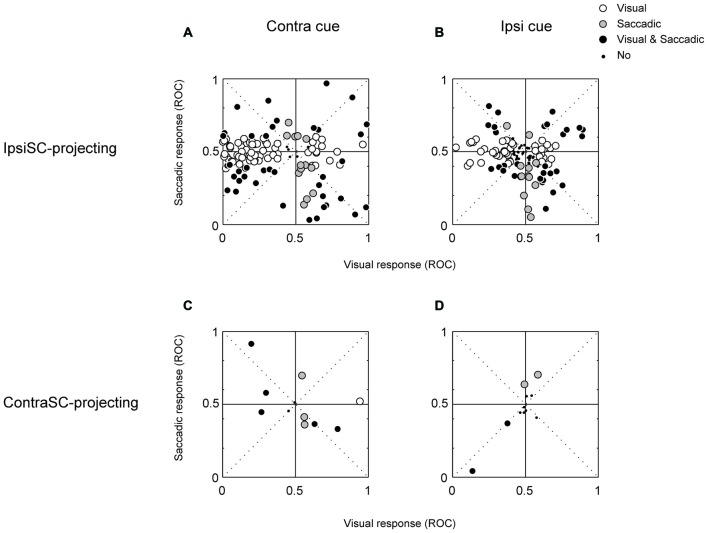
**The relationship between visual (abscissa) and saccadic (ordinate) responses of ipsiSC-projecting (A,B)** and contraSC-projecting **(C,D)** SNr neurons during memory 1DR. Receiver operating characteristic (ROC) plots for individual neurons were calculated by comparing visual or saccadic response with baseline activity in contralateral **(A,C)** and ipsilateral **(B,D)** saccade trials. The ROC value below (above) 0.5 indicates the inhibitory (excitatory) response. Opened circles: significance in visual response but not in saccadic response (**A**: 65/124, **B**: 44/124, **C**: 1/13, **D**: 0/13). Gray circles: significant in saccadic response but not in visual response (**A**: 13/124, **B**: 13/124, **C**: 4/13, **D**: 3/13). Black circles: significant both in visual and saccadic response (**A**: 39/124, **B**: 31/124, **C**: 5/13, **D**: 2/13). Small dots: neither visual nor saccadic response was significant (**A**: 7/124, **B**: 36/124, **C**: 3/13, **D**: 8/13).

These results suggest that “immediate action” and “delayed action” are controlled by different group of SNr neurons. To examine this possibility, we quantified the excitatory and inhibitory responses in each of the visual and saccadic response periods for memory-1DR (Figure 5). Each data point indicates a single SNr neuron. Data are shown separately for ipsiSC-projecting SNr neurons (top) and contraSC-projecting SNr neurons (bottom). Let us focus on ipsiSC-projecting SNr neurons which have been described above.

Figures 5A,B suggest that ipsiSC-projecting SNr neurons are separated into two groups based on their visual responses. Neurons inhibited by the visual cue (i.e., data points on the left side, post-cue ROC < 0.5) showed little change in activity before the delayed saccade: saccadic ROCs, on average, were not significantly separated from 0.5 (*t* test, *P* = 0.9). Majority of them (65/124) showed only visual responses (open circle). These features are clearer for contralateral saccades (Figure 5A) than ipsilateral saccades (Figure 5B). These neurons thus belong to “immediate type”. In contrast, SNr neurons excited by the visual cue (post-cue ROC > 0.5) tend to show inhibitions before the delayed saccade (i.e., data points in the right-bottom square): saccadic ROCs, on average, were significantly lower than 0.5 (*t* test, *P* = 0.02). These neurons thus belong to “delayed type”. These results suggest that ipsiSC-projecting SNr neuros are separated into two groups: “immediate type” and “delayed type”.

**Figure 6:**
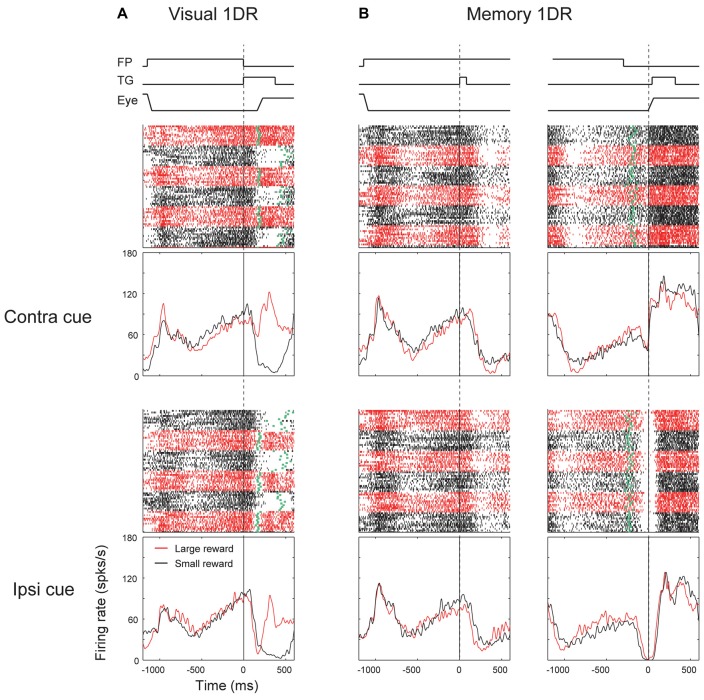
**Responses of an contraSC-projecting SNr neuron in visual 1DR (A)** and memory 1DR **(B)**. The same format as in Figure 3.

These different response patterns may arise from different neuronal circuits. As described before (Table 1), a minority of SNr neurons projected to the contralateral SC. Their response patterns were variable (Figure 5, bottom). In particular, there was no clear sign of “immediate type” or “delayed type”. However, some of them showed striking responses, as shown in Figure 6. The neuron was strongly inhibited before the saccade to the ipsilateral (not contralateral) side in memory-1DR (Figure 6B, lower right). This activity matches the scheme shown in Figure 1: inhibition of contraSC-projecting SNr neuron facilitates ipsilateral saccade. However, this contraSC-projecting SNr neuron showed several other responses which may affect saccades in different ways. In both visual-1DR and memory-1DR, the neuron was inhibited by the visual cue. In visual-1DR, the response was modulated by the expected reward value or the saccade onset (green dots).

### The Motivational Signal in SNr-SC Pathway

**Figure 7:**
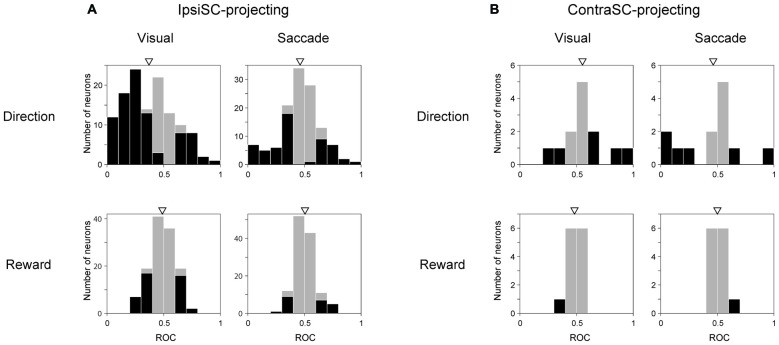
**Neuronal discriminability for saccade direction and reward amount measured by ROC.** ROC for individual neurons were calculated by comparing visual (left panels in **A,B**) or saccadic (right panels in **A,B**) response in contralateral with ipsilateral saccade trial for direction (upper row), and by comparing responses in large-rewarded with small-rewarded trials for reward discriminability (lower row). Black bars indicate neurons with statistically significant discrimination assessed by Wilcoxon rank-sum test (*P* < 0.05). The ROC value below (above) 0.5 in “Direction” and “Reward” indicates more inhibition in contralateral (ipsilateral) saccade trial and large- (small-) rewarded trial, respectively. An arrow indicates the mean of the ROC values (**A**, Direction Visual (top-left): 0.37, Direction Saccade (top-right): 0.46, Reward Visual (bottom-left): 0.49, Reward Saccade (bottom-right): 0.51, **B**, Direction Visual (top-left): 0.55, Direction Saccade (top-right): 0.46, Reward Visual (bottom-left): 0.48, Reward Saccade (bottom-right): 0.50). The mean ROC is significantly different from 0.5 only in directional discriminability in ipsiSC-projecting neurons (**A**, Direction Visual (top-left): *P* = 1.2 × 10^−9^, Directional Saccade: *P* = 0.02, *t* test).

Fundamental aspects of goal-directed saccades are direction and motivation. To quantify the discriminability of direction and reward information, we calculated ROC for direction bias by comparing the ipsilateral condition with the contralateral condition, and ROC for reward bias by comparing the large reward condition with the small reward condition. We performed this analysis separately for the visual and saccadic periods in memory-1DR. IpsiSC-projecting SNr neurons were preferentially modulated by direction rather than reward in both visual and saccade periods (Figure 7A). In particular, the ROC distribution of directional bias in the visual period was heavily skewed toward 0, indicating strong directional bias toward the contralateral direction (Figure 7A, top-left). Among 124 ipsiSC-projecting SNr neurons, 56% of neurons were significantly biased to contralateral (among them, 90% were immediate-type and 1% were delayed-type) and 15% of neurons were significantly biased to ipsilateral direction (among them, 53% were delayed-type neurons and 10% were immediate-type). On the other hand, these ipsiSC-projecting SNr neurons were less modulated by reward in both time periods (significant modulation in 34% and 18% of neurons for visual and saccade periods, respectively). Some of the contraSC-projecting SNr neurons showed directional preference, especially in the saccade period, but as a population, there was no clear contralateral preference (Figure 7B, top). They rarely showed reward-based preference (Figure 7B, bottom).

**Figure 8:**
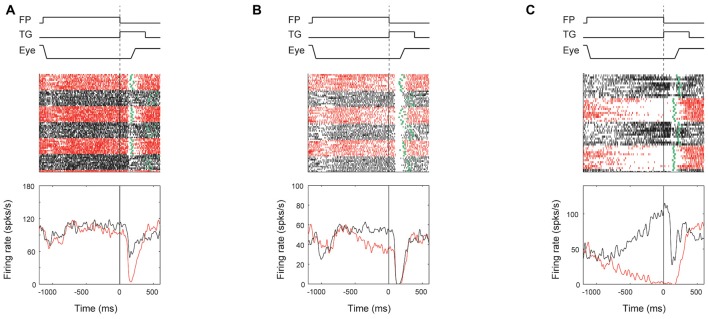
**Reward effect of three examples of ipsiSC-projecting SNr neurons in visual 1DR.** The same format as in Figure 3, except for showing the activities only in contralateral saccade trials. The ROC value of neuron’s activity (large reward vs. small reward) in pre-cue period is 0.28 (*P* = 0.002, rank-sum test) for **(A)**, 0.19 (*P* = 6.5 × 10^−6^) for **(B)**, and 0.01 (*P* = 1.6 × 10^−9^) for **(C)**. The ROC value of neuron’s activity in post-cue period is 0.13 (*P* = 6.4 × 10^−8^) for **(A)**, 0.39 (*P* = 0.09) for **(B)**, and 0.19 (*P* = 1.7 × 10^−4^) for **(C)**.

Although ipsiSC-projecting SNr neurons showed strong preference to encoding directional information, some of them encoded reward information as well. To analyze the reward effect on ipsiSC-projecting SNr neurons’ activity and behavior bias, we focused on the response in visual-1DR, because their visual as well as anticipatory response should influence saccade initiation directly. Figure 8 shows typical patterns of reward effect for ipsiSC-projecting SNr neurons during visual-1DR. The neuron in Figure 8A, which was recorded from monkey D, showed clear reward effect in visual response. The neuron was clearly inhibited by the visual cue indicating a large reward, but less inhibited by the cue indicating a small reward. Another neuron recorded from monkey N (Figure 8B) was strongly inhibited by the visual cue, whether it indicated a large or small reward. However, its activity was clearly modulated before the cue onset: the activity decreased before cue onset during a block of trials in which a large reward was delivered after the saccade to the contralateral target (red curve). This pre-cue inhibition would facilitate the contralateral saccades, which in fact occurred consistently (Figure 8B, green dots). The third neuron in monkey N (Figure 8C) showed a stronger activity bias in the pre-cue period: inhibition during the contralateral large-rewarded block (red) vs. excitation during the ipsilateral large-rewarded block (black). Due to this pre-cue activity bias, the contralateral saccade would be facilitated if it leads to a large reward, but suppressed if it leads to a small reward, which actually occurred (Figure 8C, green dots).

**Figure 9:**
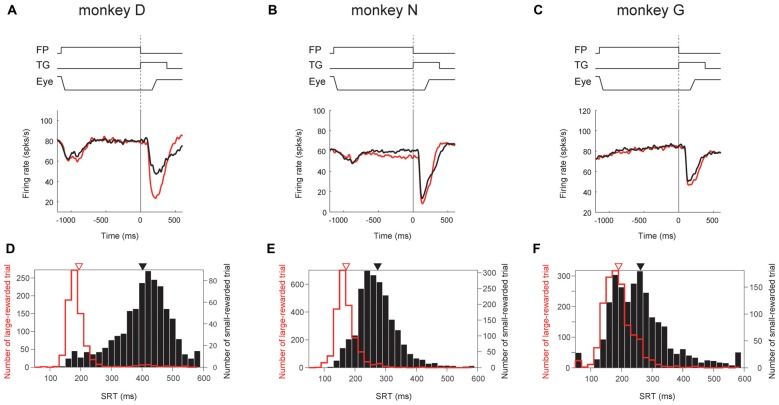
**Correlation between ipsiSC-projecting SNr neurons’ reward effect and saccadic reaction time (SRT) bias across monkeys.** Population averaged responses of the immediate-type neurons (opened circles in Figure 5A) in visual 1DR are shown in SDFs aligned on the cue onset for three monkeys (*N* = 12, 35 and 26 for **A,B,C**, respectively). Red and black SDFs indicate the averaged response in large- and small-rewarded trials, respectively. The distributions of SRTs in large- (red bars) and small- (black bars) rewarded trials are shown in **(D–F)**. An arrow indicates the mean of the SRTs: 195 (red) and 402.4 (black) ms for **(D)**, 171 (red) and 274 (black) ms for **(E)**, and 191 (red) and 262 (black) ms for **(F)**. The mean SRTs are significantly different between large- and small-rewarded trials for three monkeys (*P* ≈ 0 for **D,E**, *P* = 1.5 × 10^−12^ for **F**, *t* test).

We also found that the degree of reward effect was variable across monkeys in terms of their neuronal activity as well as their saccade behavior (Figure 9).

Among the three monkeys, ipsiSC-projecting SNr neurons in monkey D showed the largest reward effect in the post-cue period (large vs. small, *P* = 1.2 × 10^−4^, paired *t* test, ROC = 0.29; Figure 9A). SNr neurons in monkey N showed weaker reward effects, mainly in the pre-cue period (*P* = 3.5 × 10^−4^, paired *t* test, ROC = 0.39; Figure 9B). SNr neurons in monkey G showed a very weak reward effect in the post-cue period (Figure 9C). These differences in the SNr-neuronal activity were correlated with the monkeys’ saccade behaviors: the differences in the saccade reaction time were largest in monkey D, followed by monkey N, and G (Figure 9, bottom).

## Discussion

We identified ipsiSC- and contraSC-projecting SNr neurons by antidromically stimulating both sides of SC. Consistent with previous anatomical findings, a majority of antidromically activated SNr neurons had uncrossed connections to SC (Jayaraman et al., 1977; Beckstead et al., 1981; Gerfen et al., 1982; Huerta et al., 1991). Many of them showed phasic inhibitions of activity immediately after the visual stimulus was presented (immediate-type SNr neurons). In contrast, another group of neurons were excited by visual stimulus presentation, but showed inhibitions before a saccade occurred later (delayed-type SNr neurons). Since SNr sends strong inhibitory projections to SC, the inhibition of SNr neurons would cause a disinhibition of SC neurons, thus facilitating saccades. The excitation of SNr neurons would cause an inhibition of SC neurons, thus suppressing saccades. These results indicate that the signals from the ipsilateral SNr compete with each other in SC to facilitate or suppress a saccade immediately after a visual stimulus is detected. In other words, two separated populations of SNr neurons contribute to a behavioral bias toward an immediate or delayed action by sending antagonistic signals to the ipsiSC.

**Figure 10:**
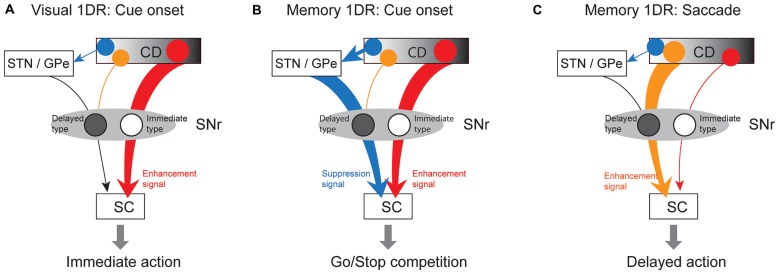
**Hypothetical scheme showing separate circuits in basal ganglia (BG) that control saccades in different contexts. (A)** In visual 1DR, the immediate initiation of a saccade after cue onset is caused by the cue-induced inhibitory response of “immediate-type” SNr neurons, which leads to a disinhibition of SC saccadic neurons. This is mediated by the direct pathway (red arrow; CD-SNr-SC). **(B)** In memory 1DR, withholding an immediate saccade after cue onset is caused by the cue-induced excitatory response of “delayed-type” SNr neurons, which leads to an enhanced inhibition of SC saccadic neurons. This is mediated by the indirect pathway (blue arrow; CD-GPe/subthalamic nucleus (STN)-SNr). **(C)** To initiate a delayed saccade in memory 1DR, pre-saccadic inhibition of “delayed-type” SNr neurons causes a disinhibition of SC saccadic neurons. This is mediated by the direct pathway (orange arrow). “Immediate-type” and “delayed-type” SNr neurons receive the direct pathway inputs from different populations of CD neurons (see “Discussion” Section).

How does SNr use these antagonistic signals to perform goad-directed eye movement? In visual-1DR task, the robust phasic inhibition of immediate-type SNr neurons would urge SC neurons to initiate a saccade immediately (Figure 10A). Almost all (97%) of immediate-type SNr neurons were consistently inhibited by visual stimuli not only in memory-1DR but also in visual-1DR, while a less proportion (76%) of delayed-type SNr neurons were excited in visual-1DR. The robust inhibitory responses of immediate-type neurons may promote immediate saccades. In memory-1DR task, cue onset caused the competition between two antagonistic signals in SC (Figure 10B). The cue stimulus briefly inhibited immediate-type SNr neurons, which may break gaze fixation reflectively, but excited delayed-type SNr neurons, which may suppress the fixation break. Before the saccade, immediate-type SNr neurons are insensitive. Instead, the decrease in activity of delayed-type SNr neurons would facilitate the delayed saccade (Figure 10C).

The excitation of delayed-type SNr neurons may be caused by the excitatory input from the subthalamic nucleus (STN) or the globus pallidus external (GPe) segment (Figure 10). It was shown previously that some STN neurons were phasically excited if a visual stimulus appeared so that the monkey had to switch from the prepared saccade to the other saccade (Isoda and Hikosaka, 2008). The main action of these STN neurons may be to promote behavioral switch by suppressing the prepared saccade. A similar mechanism may be used to suppress the saccade toward the target cue in memory-1DR task in our experiment. Such a context-dependent signal of Delayed-SNr neurons would enable flexible behaviors.

GPe may also contribute to the excitatory response of delayed-type SNr neurons. The anterior part of CD—caudate head (CDh) and caudate body (CDb)—contains many neurons that are excited by visual stimuli (Rolls et al., 1983; Hikosaka et al., 1989b; Kim and Hikosaka, 2013). The visual signal may be sent to SNr through the indirect pathway that is mediated by GPe (Parent and De Bellefeuille, 1983; Smith and Bolam, 1991). This would lead to an excitation (i.e., disinhibition) of SNr neurons, since both CD-GPe and GPe-SNr connections are inhibitory (Smith and Bolam, 1990; Kita, 2007).

The inhibition of delayed-type SNr neurons before the delayed saccade may be caused by another signal from CD. Many neurons in CDh or CDb are excited before delayed saccades (Lau and Glimcher, 2007), especially memory-guided saccades (Hikosaka et al., 1989a). If this pre-saccadic signal is sent to SNr neurons directly, they would be inhibited before delayed memory-guided saccades, which indeed was shown previously (Hikosaka and Wurtz, 1983a).

These arguments raise an interesting question about the BG circuits. It has been suggested that the direct and indirect pathways work together: the direct pathway initiates an action, while the indirect pathway suppresses other actions (Graybiel, 2000). This is a popular hypothesis about the BG mechanism of action selection. Our arguments above suggest another function of direct/indirect pathways, as suggested previously (Hikosaka et al., 2000): suppress an action first (with the indirect pathway), and later release the action (with the direct pathway). This mechanism may be important in real life, because we often need to withhold an action until it becomes beneficial.

In contrast, the behavior of immediate-type SNr neurons looks much simpler. They were inhibited phasically by a visual stimulus on the contralateral side, which would lead to a disinhibition of SC neurons, thus facilitating a saccade to the stimulus immediately. This inhibition is likely to be caused by the direct input from CD (Precht and Yoshida, 1971; Graybiel, 1990; Figures 10A,B). But they do not change their activity with the delayed saccade. These results may suggest that immediate-type SNr neurons receive inputs only from the direct pathway. This is unlikely, however, for the following reason.

We reported previously that many SNr neurons projecting to the ipsiSC were inhibited by visual objects (Yasuda et al., 2012), similarly to immediate-type SNr neurons in this study. Such SNr neurons receive visual signals from the tail of CD (CDt) via both the direct and indirect pathway (Yasuda and Hikosaka, 2015). However, the indirect pathway is active only after the reward value of the visual target has been fixed for a long time (i.e., stable reward value). When the reward value changes flexibly (as in our 1DR task), only the direct pathway is active so that visual signals cause pure inhibitions in SNr neurons. This may be what occurred in immediate-type SNr neurons: the indirect pathway does affect immediate-type SNr neurons, but only when the visual inputs have stable values, which was not the case in 1DR task.

The above discussions suggest that delayed-type SNr neurons receive inputs from CDh or CDb, while immediate-type SNr neurons receive inputs from CDt. In fact, our recent studies have suggested that both CDh and CDt have separate downstream circuits aiming at SC (Hikosaka et al., 2014; Yasuda and Hikosaka, 2015). The separate circuits process reward values of visual objects differently: flexibly by CDh-circuit vs. stably by CDt-circuit. These results together suggest that two types of goal-directed eye movements, delayed and immediate saccades, may be controlled by the two parallel circuits: CDh-SNr-SC circuit and CDt-SNr-SC circuit.

In addition to uncrossed SNr-SC connection, we also examined the signal processing in the crossed connection. Neuronal signals in the crossed SNr-SC connection was examined by Jiang et al. (2003) using anesthetized cats (Jiang et al., 2003). They reported that all crossed SNr neurons showed excitatory visual responses, which would suppress saccades away from the goal. We found similar neuronal activity in several neurons. However, we also found inhibitory responses when the monkey withheld the saccade in memory-1DR task (Figure 6). Such context-dependent signal might appear when the animal is aiming at a goal. Jiang et al. (2003) also described the low spontaneous firing of crossed SNr neurons. However, we found no clear differences in baseline activity between crossed and uncrossed SNr neurons. This discrepancy also may be due to the difference in arousal level. During recording of crossed SNr neurons, we sometimes detected that the neuronal firing became slow and irregular when the monkey became drowsy. Because of the sparseness of the crossed SNr-SC connection, its function is still unclear in the monkey.

## Author Contributions

MY and OH designed research; wrote the article. MY performed research; analyzed data.

## Funding

This research was supported by the Intramural Research Program at the National Institutes of Health, National Eye Institute.

## Conflict of Interest Statement

The authors declare that the research was conducted in the absence of any commercial or financial relationships that could be construed as a potential conflict of interest.
